# The Toxicity of Lead and Lead‐Free Perovskite Precursors and Nanocrystals to Human Cells and Aquatic Organisms

**DOI:** 10.1002/advs.202415574

**Published:** 2025-02-10

**Authors:** Immacolata Maietta, Clara Otero‐Martínez, Sabela Fernández, Laura Sánchez, África González‐Fernández, Lakshminarayana Polavarapu, Rosana Simón‐Vázquez

**Affiliations:** ^1^ CINBIO Grupo Inmunología Universidade de Vigo Vigo 36310 Spain; ^2^ Inflammatory and Infectious Diseases and Immune Disorders Group, Galicia Sur Health Research Institute (IIS Galicia Sur) SERGAS‐UVIGO Spain; ^3^ CINBIO Materials Chemistry and Physics Group Universidade de Vigo Campus universitario Vigo 36310 Spain; ^4^ Department of Physical Chemistry CINBIO Campus Universitario As Lagoas‐Marcosende Universidade de Vigo Vigo 36310 Spain; ^5^ Department of Zoology Genetics and Physical Anthropology Faculty of Veterinary Science Universidade de Santiago de Compostela Lugo 27002 Spain; ^6^ Preclinical Animal Models Group Health Research Institute of Santiago de Compostela, (IDIS) Santiago de Compostela Spain

**Keywords:** cytotoxicity, environment, halide perovskites, human health, perovskite nanocrystals

## Abstract

Halide perovskites have emerged at the forefront of semiconductor materials for photovoltaic and light‐emitting devices. However, long‐term stability and toxicity are the major barriers to their commercialization. In particular, the toxicity of lead (Pb) and its environmental impact have prompted the exploration of lead‐free alternatives like tin (Sn) and bismuth (Bi). Herein, a cytotoxicity study of Pb‐based perovskite (CsPbBr_3_ and CsPbI_3_) and a lead‐free Cs_2_AgBiBr_6_ nanocrystals in lung and liver cell lines and in blood cells are reported, revealing that both Pb and Bi exhibit mitogenic effects and oxidative stress in liver cells, cytotoxicity in pulmonary cells and a dose‐dependent hemolytic effect, raising concerns about their potential pulmonary‐hepato‐hemotoxicity. The zebrafish embryo tests reveal SnBr_2_ displays a notably safer environmental impact profile at elevated concentrations, while Pb and Bi exhibit dose‐dependent toxicity. This study provides compelling evidence that Sn compounds demonstrate a safer toxicity profile in both human cells and aquatic organisms under the studied conditions, supporting their potential as a more environmentally friendly alternative. In contrast, Bi compounds exhibit a toxicity profile similar to Pb perovskites, warranting caution in its use as a substitute. These findings guide future research and regulatory efforts in the safety and sustainability of perovskite nanocrystals.

## Graphical abstract

The cytotoxicity of Lead‐ and Lead‐free halide perovskite precursors and colloidal nanocrystals for human cells (lungs, liver, and blood) and aquatic organisms (zebrafish) is reported. The study finds that Sn displays a notably safer environmental impact profile, whereas Bi and Pb exhibit cytotoxic effects and oxidative stress in liver and pulmonary cells and a dose‐dependent hemolytic effect.

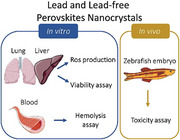

## 1 Introduction

Lead (Pb) halide perovskite nanocrystals (NCs) are a newly emerged family of semiconductor materials with the formula APbX_3_, where A is a monovalent cation (methylammonium, cesium, or formamidinium), and X is a halide (chloride, bromide, or iodide).^[^ ^1^ ^]^ The characteristics of these semiconductors (interesting optical properties, low‐cost, tunable bandgap depending on composition or dimension, defect‐tolerance, near‐unity photoluminescence quantum yield (PLQY)), together with facile synthesis (not requirement of inert conditions and easy to crystallize)^[^ ^2^ ^]^ have led them to rapid progress for photovoltaics, light‐emitting diodes (LEDs), lasers and beyond.^[^ ^3^ ^]^ However, the potential of these materials is accompanied by concerns about the toxicity induced by lead (Pb) and its effect on the environment and human health. Substituting the Pb‐cation with more environmentally friendly metals such as tin (Sn) or bismuth (Bi) has become a popular alternative to mitigate its toxicity.^[^ ^4^ ^]^ Despite significant progress in the development of lead‐free perovskite NCs, photovoltaic devices have not managed to achieve the same efficiency levels as those obtained with Pb‐based perovskites. While intensive research is being carried out on Pb‐free perovskites, it is still not clear whether the alternatives are non‐toxic or in what way they represent a significant improvement with respect to environmental and health concerns.^[^ ^5^ ^]^

The ionic nature of perovskites makes them susceptible to moisture and water, which can lead to its degradation into salts of PbX_2_ and AX, along with its consequent dissolution.^[^ ^5^, ^6^ ^]^ This makes rainwater an access route and transmission medium for perovskites to enter into the surrounding environment. Despite the low solubility of Pb, Sn, and Bi salts in water, the acidic pH of rainwater, resulting from gas emissions, favors their dissolution in both water and soil. Therefore, the influence of these ions released from perovskite NCs on the environment cannot be ignored and has already started to be studied.^[^ ^6^, ^7^ ^]^ Benmessaoud et al. have characterized, using multiple assays, the damaging effects of MAPbI_3_ perovskite on several human cell lines and emphasize that it is not only due to the presence of lead, but also significantly involves the MA cation. Those results have also been corroborated in some plants, affecting their growth.^[^ ^6^, ^8^ ^]^ On the other hand, Babayigit et al. tested and compared the pathology and genotoxicity induced by PbI_2_ and its most common substitute, SnI_2_, in a zebrafish as an organism model. They found that due to the natural instability of SnI_2_, the tin salt has a higher lethal response than PbI_2_ at equal concentrations.^[^ ^5a,b^ ^]^

However, most of the previous studies were mainly focused on bulk perovskites and their precursor salts, while the field of NCs is still quite unexplored regarding their impact on health and environment. There is a lack of studies on the impact of NCs on human cells representative of the most relevant organs in accidental exposure, such as the lungs, the liver or the red blood cells. Considering the high surface/volume ratio of the NCs, which have a lateral size of less than 15 nm, and a ligand (oleylamine and oleic acid)‐stabilized surface,^[^ ^9^ ^]^ the main question is whether these NCs will have any influence on human toxicity and a significant environmental impact.

**Figure 1:**
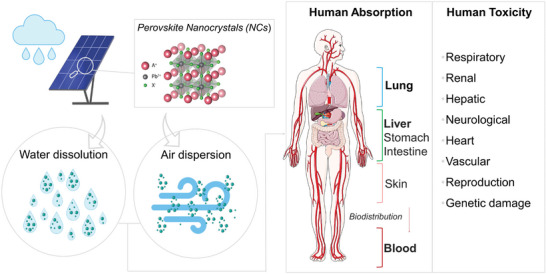
Illustrative diagram representing the potential human body's absorption and distribution of lead (Pb) halide perovskite nanocrystals (NCs). Colloidal perovskite NCs can disperse into the air or dissolve into water, which allows them to enter the body through water intake or inhalation during handling. NCs can be absorbed through the respiratory, gastrointestinal, or dermal systems, and their distribution via blood vessels enables them to reach various organs and other parts of the body. These materials can accumulate in different tissues, leading to systemic toxicity and posing a risk to human health. Lung and liver are the two most potentially affected organs due to their tendency to accumulate intake particles. For this reason, human lung and hepatic cell lines were selected for in vitro toxicological studies. Additionally, the potential hemolytic effect of the NCs was also tested in whole human blood samples.

In addition, the colloidal nature of these NCs allows them to disperse into the air, so these metals could access the body not only through water intake, but also directly through inhalation during handling and throughout their life cycle. Thus, they could reach different organs, such as the lungs or bloodstream, and be further biodistributed to other parts of the body (**Figure** **1**).^[^ ^6^, ^7^, ^10^ ^]^ The possibility of direct exposure to the lungs and bloodstream makes the use of cells from both sources essential for in vitro risk assessment studies, as well as from other tissues in which NCs can accumulate, such as the liver. However, although in vitro studies can provide information about cell toxicity, these results must be complemented with in vivo experiments to gain a broader understanding of the biodistribution, metabolism, and systemic toxicity of NCs.

This approach is necessary to fully comprehend the potential risks that these nanomaterials could pose to human health. Here, we compared the effects of Pb‐based and Pb‐free perovskite NCs, as well as the precursor salts employed for synthesis of perovskite NCs and fabrication of solar cells and LEDs, in several human cellular models: including lung epithelial cell lines (A549 and NCI‐H460), liver cell line (HEPG‐2), and fresh human blood erythrocytes. Finally, in vivo studies of the exposure to perovskite NCs were conducted using zebrafish embryos as a model organism. Our results support the use of Sn as a more environmentally friendly alternative to Pb, while Bi compounds, specifically Bi(Ac)₃, should be more carefully evaluated, because they showed similar toxicity to Pb in both, human cells and zebrafish embryos.

## 2 Results and Discussion

### 2.1 Effect of Perovskite NCs and their Precursors on Human Lung and Liver Cell Lines

**Figure 2:**
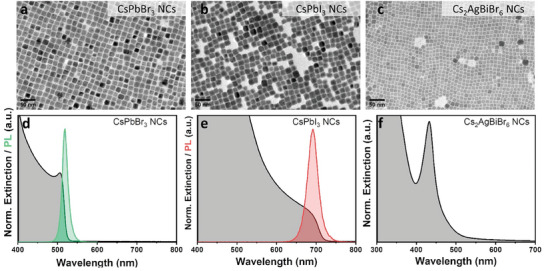
a–c) TEM images of colloidal dispersions of CsPbBr_3_ (a), CsPbI_3_ (b), and Cs_2_AgBiBr_6_ nanocrystals (NCs) (c). d–f) Extinction and PL spectra of colloidal dispersions of CsPbBr_3_ (d), CsPbI_3_ (e), and Cs_2_AgBiBr_6_ (f) NCs.

We tested two Pb‐based (CsPbBr_3_ and CsPbI_3_) and one Pb‐free (Cs_2_AgBiBr_6_) perovskite NCs along with their precursors (Bi(Ac)_3_, PbI_2_, PbBr_2_, and Cs_2_CO_3_) to compare the potential toxicity of Pb and Bi on the lung and liver cell lines, as these are potential target organs after accidental exposure through inhalation or ingestion. For comparison, SnBr_2_ was also tested as a common source of Sn in lead‐free perovskite NC synthesis. The NC colloidal solutions were synthesized according to previously reported procedures (see experimental section for more details). The TEM images of the NCs are shown in **Figure** **2**a‐c. In all the cases, the NCs exhibit the typical cubic morphology with a size of ≈10 nm. The NCs show the typical absorption features with green and red PL emission for CsPbBr_3_ and CsPbI_3_ NCs, respectively, while Cs_2_AgPbBr_6_ is non‐luminescent (Figure 2d–f). The NC colloidal solutions were purified by precipitation with antisolvent and then dried to obtain them in powder form, and the different cell lines were exposed to the NCs (see experimental section for more details).

To determine the potential health hazard associated with the individual components of Cs_2_AgBiBr_6_, CsPbI_3_, and CsPbBr_3_ perovskite NCs, we also tested the four NC precursors Bi(Ac)_3_, PbI_2_, PbBr_2_ and Cs_2_CO_3_, and SnBr_2_ for comparison. First, we selected two different lung epithelial cell lines, A549 and NCI‐H460, and the liver cell line HEPG‐2 as model cell targets. The effect of the NCs and their precursors on the cell viability was tested at different concentrations (0.01 – 500 µm and 1 mm) at 96 h, using an MTS (tetrazolium salt) test. The components and the NCs showed a limited solubility in the cell medium at high concentration (Figure S1, Supporting Information), except for Cs_2_CO_3_ and PbBr_2_ that were the most soluble compounds.

**Figure 3:**
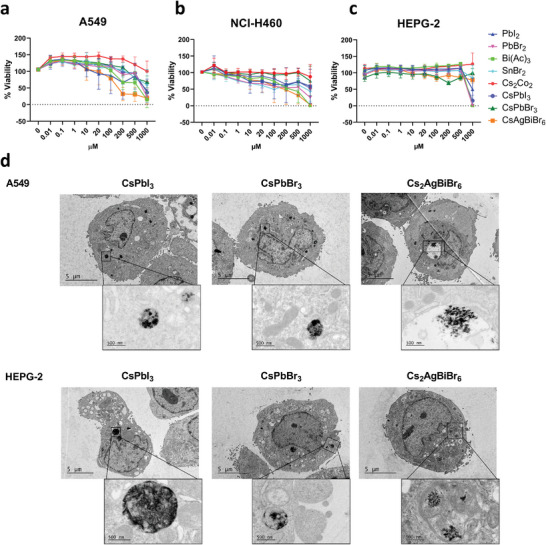
Cytotoxicity induced by perovskite nanocrytals (NCs) precursors and perovskite NCs. Changes in cell viability of A549 (a), NCI‐H460 (b), and HEPG2 (c) cells incubated for 96 h with different concentrations (0.01 – 500 µm and 1mm) of four different halide perovskite NC precursors: Bi(Ac)_3_, PbI_2_, PbBr_2_ and Cs_2_CO_3_; and three NCs: Cs_2_AgBiBr_6_, CsPbI_3,_ and CsPbBr3. SnBr_2_ was also tested for comparison. Transmission electron microscopy (TEM) images (d) of A549 and HEPG‐2 cell lines in the presence of perovskite NCs (Cs_2_AgBiBr_6_, CsPbI_3,_ and CsPbBr_3_). The cells showed the presence of vesicles with NCs accumulation (black aggregates) in the cell cytoplasm but not in the nucleus. Magnification 800x (5 µm), 1000x (5 nm).

After 96 h of incubation, NCs and all precursors, except Cs_2_CO_3_, induced cell toxicity at ≥100 µm in both pulmonary cell lines (**Figure** **3**a,b), being the A549 cell line more sensitive than the NCI‐H460 one (Table S1, Supporting Information, half inhibitory concentration (IC50) values). Interestingly, Cs_2_CO_3_ was the only compound that did not alter the cell viability of both cell lines at any of the concentrations tested. A dose‐dependent effect was observed from 100 µm to 1 mm, while no significant changes were observed at the lowest concentrations. Conversely, only CsPbI_3_, and Cs_2_AgBiBr_6_ NCs to a lesser extent, along with their precursors PbI_2_ and Bi(Ac)_3_, induced toxicity in the liver cell line (Figure 3c) after 96 h of incubation at the highest concentration tested (1 mm), while not significant changes were detected with the other compounds.

SnBr_2_ was less toxic than the Pb and Bi precursors in both lung and liver cells, except in the NCI‐H460 lung cell line, where Sn precursors showed a level of toxicity like the observed with the Pb precursors (Figure 3; Table S1, Supporting Information). The compounds' toxicity was further analyzed using a real‐time cell analyser (RTCA), which measures changes in cell impedance due to cell proliferation or detachment, expressed as a cell index (CI). This analysis was conducted on the adherent A549 and HEPG‐2 cell lines (Figure S2, Supporting Information). Notably, only the Bi(Ac)_3_ precursor caused a relevant decrease in the CI on both cell lines. However, while this effect was time‐dependent in the A549 cells, HEPG‐2 cells showed recovery from 48 to 96 h. Differences in the results of these two tests can be attributed to the parameters that each method assesses: while the MTS assay measures cell metabolism, the RTCA system measures cell adherence.

Previous studies have reported such differences, with both assays offering complementary insights into cell changes in response to stimuli.^[^ ^11^ ^]^ The apparent lower toxicity observed in the RTCA assay at 96 h may be due to the system's reduced sensitivity to detect differences at high cell densities. To further investigate whether the changes in cell viability were associated with cell death, cells were incubated for 96 h with the NCs and precursors at 500 µm, then stained with calcein‐acetoxymethyl (calcein‐AM, green fluorescence) and ethidium (red fluorescence) to distinguish live from dead cell populations (Figure S3, Supporting Information). The fluorescence images demonstrated that all compounds induced cell death to varying degrees. In line with the MTS assay results (Figure 3), Bi(Ac)_3_ at 500 µm significantly reduced cell density in both lung epithelial cell lines, corresponding with increased cell death. This effect was not observed in the hepatocarcinoma‐derived HEPG‐2 cell line at the tested concentration, as seen also for the MTS assay (Figure 3c).

The viability in all cell lines showed a relevant decrease when the NCs were tested at the highest doses (500 µm and 1 mm) after 96 h of exposition, except for CsPbBr_3_ in the HEPG‐2 and NCI‐H460 cells (Figure 3). This could be likely due to a Troyan effect of the metal and precursor ions released from the NCs following cell uptake, associated with a higher cell internalization of the NCs in comparison with the precursors.

To confirm the internalization of perovskite NCs (Cs_2_AgBiBr_6_, CsPbI_3,_ and CsPbBr_3_) into the cells, both, the lung A549 and the liver HEPG‐2 cell lines were incubated with the NCs at 100 µm for 96 h and subsequently analyzed by transmission electron microscopy (TEM) (Figure 3d). In the TEM images, the NCs or their degraded products appeared as black dot aggregates enclosed within vesicles in the cytoplasm, with no observed effects on the nuclear structure.

Interestingly, the NCs induced higher cytotoxicity than the precursors, except for Bi(Ac)_3_, as seen by both, the MTS and RTCA assays (Figure 3; Figure S2, Supporting Information, respectively). Among the precursors, Cs_2_CO_3_ exhibited the lowest toxicity, while Bi (Bi(Ac)_3_) showed higher or similar toxicity than that induced by Pb (PbI_2_, PbBr_2_) in the lung and liver epithelial cell lines. In general, Sn (SnBr_2_) was less toxic than Pb and Bi. Pb has been classified as a renal carcinogen in rodents.^[^ ^12^ ^]^ In humans, most Pb accumulates in bones but also causes chronic damage in the liver, kidneys, and other organs such as the nervous, cardiovascular, and reproductive systems.^[^ ^13^ ^]^ Based on animal studies and some evidence in humans, the International Agency for Research on Cancer (IARC) has classified Pb as a potential carcinogen to humans.

In contrast, Bi has been considered harmless to human health and has increasingly been used as a “green” substitute for Pb in many industrial applications. Moreover, Bi is used in medical applications due to its associated biological properties, such as antimicrobial and anticancer activities.^[^ ^14^ ^]^ However, high Bi uptake can induce nephro‐, hepato‐, and neurotoxicity, among other events, which largely depends on the Bi compound used, with soluble compounds being more toxic than insoluble ones.^[^ ^14^ ^]^

In our case, Bi showed the highest toxicity toward hepatic and pulmonary epithelial cells, which could be related to the hepatoxicity described at high Bi concentrations and associated with a higher risk of inducing pulmonary toxicity.

In contrast, Sn was generally less toxic than both Pb and Bi. Inorganic tin is typically regarded as safe due to its low solubility and limited bio absorption. However, chemical and biochemical methylation can produce toxic organic Sn derivatives, making it advisable to avoid high exposure levels.^[^ ^15^ ^]^ Recent studies on Sn‐containing halide perovskites also suggest a safer in vivo profile for Sn compared to Pb, supporting the potential use of Sn as a safer alternative to Pb in halide perovskites.^[^ ^16^ ^]^ Biodistribution studies in mice after oral ingestion confirm the lungs and liver as two of the main organs where halide perovskites accumulate. The study also highlighted the oxidation state of the metal as a relevant factor in organ toxicity, with Sn⁴⁺ being safer than Sn^2^⁺. Interestingly, in this study, Bi^3^⁺ and Sn⁴⁺ caused lower toxicity than Pb^2^⁺ and Sn^2^⁺ in the lungs, while Pb^2^⁺ and Bi^3^⁺ were more toxic than Sn (II or IV) in the liver.^[^ ^16b^ ^]^

### 2.2 Characterization of the Haemolysis Induced by the Perovskite Precursors and NCs

**Figure 4:**
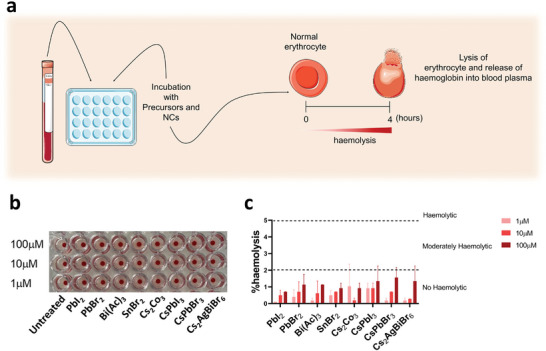
Haemolysis induced by halide perovskite nanocrystals (NCs) and their precursors. a) Graphical representation of the haemolysis analysis. The precursors (Bi(Ac)_3_, PbI_2_, PbBr_2_ and Cs_2_CO_3_) and the perovskite NCs (Cs_2_AgBiBr_6_, CsPbI_3_, and CsPbBr_3_) were used at different concentrations (1, 10, and 100 µm). SnBr_2_ was also tested for comparison. b) Cell plate after incubating blood samples with the compounds. c) Graphical representation of the percentage (%) of haemolysis after quantifying the absorbance of free haemoglobin (expressed as the mean plus standard deviation).

Among the potential haemotoxicity events induced by any nanomaterial or compound after reaching the bloodstream, haemolysis is one of the most relevant, as it can lead to conditions such as anaemia, spleen congestion, renal failure or even death if the haemolytic rate is very high.^[^ ^17^ ^]^ Some studies have shown that ≈35% of the lead dispersed into the vascular system is bound to erythrocytes, whereas only 1% reaches other compartments such as liver, kidneys, brain, lungs, and spleen.^[^ ^18^ ^]^ The high accumulation of lead in red blood cells can weaken and damage their cell membrane, causing haemolysis. This phenomenon is one of the toxicological events associated with Pb exposure, causing anemia.^[^ ^17^ ^]^ Pb is also known to affect heme synthesis by interfering with enzymes. Furthermore, blood Pb concentrations of ≥ 50 µg ×100 mL^−1^ (2.4 µm) in adults induce a significant decrease in haemoglobin levels.^[^ ^19^ ^]^ Therefore, we evaluated the potential haemolysis induced by both NCs and precursors. Haemolysis is a process derived from the rupture of red blood cells, causing the release of haemoglobin into the plasma.^[^ ^20^ ^]^ Therefore, it can be easily characterized by measuring free haemoglobin in blood samples after incubation with NCs or their precursors (**Figure** **4**a). The American Society for Testing and Materials (ASTM) has established three levels based on the percentage of haemolysis: low (<2%), moderate (2–5%) and highly haemolytic (>5%).^[^ ^21^ ^]^

The compounds were tested at three different concentrations (1, 10, and 100 µm). Higher concentrations were not tested, because the background on the absorbance interfered with the test (data not shown). Furthermore, at high concentrations, NCs dissolved in water tend to precipitate in their precursor form, making it challenging to distinguish between toxicity from the compound and that caused by precipitation. To maintain experimental accuracy, a range of lower, non‐precipitating concentrations were chosen. The results showed a low and dose‐dependent haemolytic effect (< 2%) in all analyzed samples (Figure 4b,c). No significant differences were observed between the precursors and NCs.

The haemolytic activity of Pb has been associated with reactive oxygen species (ROS) release and lipid peroxidation in the erythrocyte membrane.^[^ ^22^ ^]^ The low haemolytic effect detected in vitro at the tested concentrations for both lead‐based Perovskite NCs and the Pb precursors could be attributed to the low bioavailability of Pb ions or the kinetic time used for measurement.

### 2.3 Release of Reactive Oxygen Species on Lung and Liver Cells

**Figure 5:**
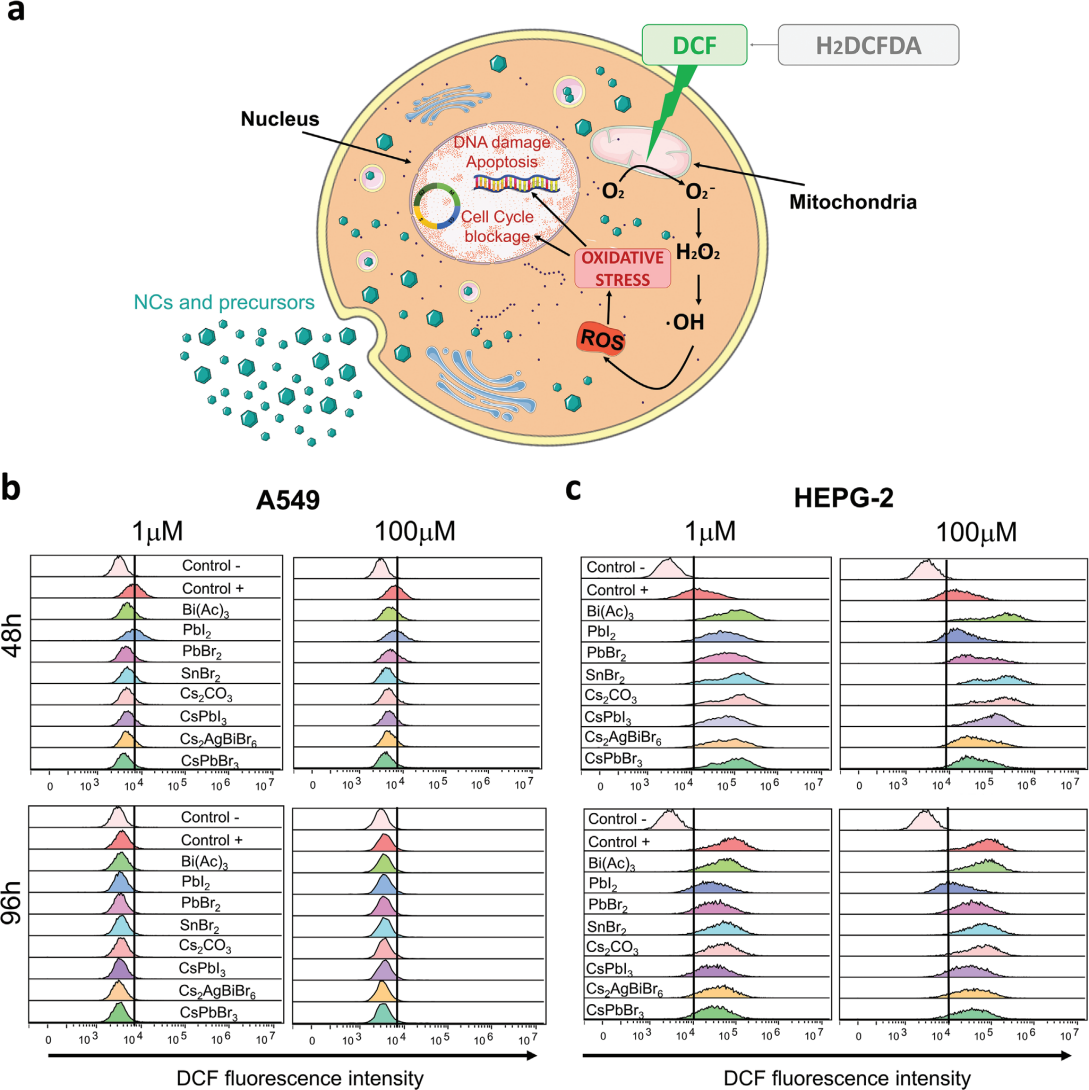
ROS release induced by halide perovskite nanocrystals (NCs) and their precursors in A549 and HEPG‐2 cell lines. a) Graphical representation of the method used to quantify cellular oxidative stress levels. (H2DCFDA: Dichlorodihydrofluorescein‐diacetate, DCF: Dichlorofluorescein) b,c) Representative histograms showing the fluorescent intensities of control cells (untreated, control ‐), phorbol 12‐myristate 13‐acetate (PMA)‐stimulated (control +) cells and cells incubated with the precursors and perovskite NCs at 1 and 100 µm after 48 and 96h. PMA failed to induce ROS in A549 cells at the times tested.

ROS are oxygen‐containing reactive molecules generated by different extracellular and intracellular reactions, which are involved in many cellular processes, like cell growth, differentiation, progression, and cell death.^[^ ^23^ ^]^ However, the accumulation of high levels of ROS, known as oxidative stress, can lead to inflammation, tumorigenesis, genome mutation, or signaling pathway instability.^[^ ^24^ ^]^ On the other hand, it is well known that Pb inactivates antioxidant enzymes such as glutathione reductase (GR), superoxide dismutase (SOD), and catalase (CAT). Redox imbalance generates neuronal, haematological, renal, hormonal, immune, and gastrointestinal injuries, and can cause hypertension and cardiovascular problems.^[^ ^18a^ ^]^ Additionally, increased free radical concentrations in cells, known as oxidative stress, lead to cellular and genetic damage, including inhibition of DNA synthesis and repair, contributing to carcinogenic processes.^[^ ^18b^ ^]^ In fact, oxidative stress is associated with the hepatotoxicity induced by Pb exposure, causing an increase in hepatic alanine aminotransferase (ALT) and aspartate aminotransferase (AST) levels in the blood.^[^ ^25^ ^]^ For this reason, we performed an assay on A549 and HEPG‐2 cell lines to test if either the precursors or the perovskite NCs were able to induce ROS production at two different concentrations (1 – 100 µm) and exposition times. Carboxy‐H2DCFDA (Dichlorodihydrofluorescein‐diacetate) is a non‐fluorescent dye used to detect ROS within cells. Phorbol‐12‐myristate‐13‐acetate (PMA) was used as a positive control in both cell lines, however, in A549 cells, it was only able to induce significant ROS levels at 96 h, but not at 48 h. Once it is inside, cellular esterases convert it into a monomeric form. Interaction with ROS, like hydrogen peroxide, triggers the oxidation of the monomer into the highly fluorescent dichlorofluorescein (DCF). This fluorescence is directly proportional to ROS levels. Interestingly, the results show that both precursors and NCs were able to induce an increase in the basal levels of ROS in the HEPG‐2 cells after 48 and 96 h of exposition to both concentrations tested (1 – 100 µm), but not in the lung derived A549 cell line (**Figure** **5**; Figure S4, Supporting Information). The precursors induced a similar release of ROS as the NCs, and contrary to the toxicological studies, no significant differences were observed for Pb, Bi, or Sn compounds.

### 2.4 Effect of NC Precursors on Zebrafish Survival

Heavy metals are known to have toxic effects on aquatic organisms, including zebrafish. For instance, metal ion shedding from dissolvable NiO and Cr_2_O_3_ nanoparticles induced hatching interference on zebrafish embryo and larvae. The toxicity was found to be concentration‐ and time‐dependent, with larvae being more sensitive to heavy metals than embryos.^[^ ^26^ ^]^ The toxicity of metal nanoparticles to living organisms is influenced by the dissolution of metal ions from the particles and their speciation, which affects their toxicokinetics and toxicodynamics. Factors such as metal valence state, solubility, biotransformation, and chemical form play key roles in determining metal speciation.^[^ ^27^ ^]^

The release of perovskite components into the environment can be transmitted to aquatic organisms, so we tested their effect using zebrafish as a model organism. The Fish embryo acute aquatic toxicity (FET) test is a critical tool for assessing the potential harm of NC precursors on early fish development.

**Figure 6:**
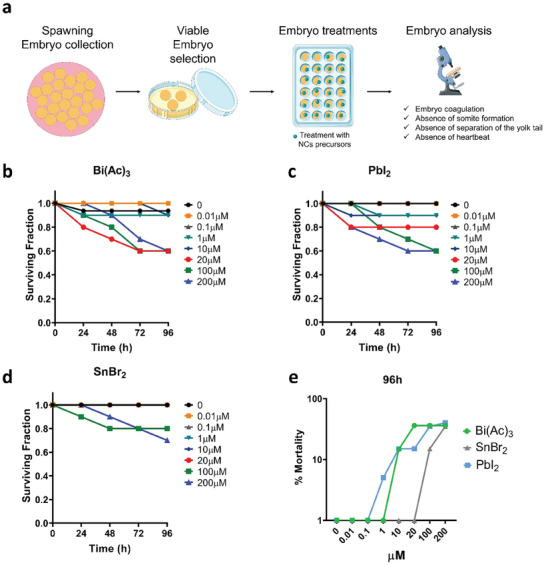
Effect of nanocrystal (NC) precursors on Zebrafish embryos. a) Schematic representation of the Fish Embryo Acute Aquatic Toxicity (FET) Test: release of eggs and sperm by aquatic animals for reproduction; fish embryos are collected and sorted for uniformity in age and size; a specific number of fish embryos are exposed to different NC precursors; the embryos are closely monitored and analyzed. b–d) Zebrafish embryo survival fraction in the presence of Bi(Ac)_3,_ PbI_2_ and SnBr_2_ at doses of 0.01 to 200 µm after an incubation period of 24, 48, 72, and 96 h. e) Concentration‐effect curve of zebrafish embryo mortality in the presence of Bi(Ac)_3_, PbI_2_ and SnBr_2_, after 96 h of incubation at doses ranging from 0.01 to 200 µm.

This method was used to evaluate the harmful effects of varying concentrations of NC precursors in zebrafish development over different time intervals, examining their toxicity profiles. To perform the FET test, the following steps are followed, as represented in **Figure** **6**a: i) after the release of eggs and sperm by aquatic animals for reproduction, the fish embryos are formed; ii) the embryos are collected and sorted for uniformity in age and size; iii) a specific number of fish embryos are exposed to different NC precursors; iv) the embryos are closely monitored and analyzed to detect any potential defect on embryo development. Due to the limited solubility of the NCs, only the precursors were analyzed. Because PbI_2_ and PbBr_2_ showed similar toxicity in vitro, only the former compound was selected for the FET test. Additionally, SnBr_2_ was studied to compare the cytotoxicity of Pb, Bi, and Sn. Zebrafish embryos aged 2–4 h post‐fertilization (hpf) were exposed to five different doses (0.01, 0.1, 1, 10, 20, 100, and 200 µm) of the NC precursors for 24, 48, 72, and 96 h. Some embryos were left untreated (as a negative control), and others were incubated with 3,4‐dichloroaniline (as a positive control). Evaluation of the controls yielded favorable results, with a survival rate in the negative control of > 90% and mortality in the positive control exceeding 70%. **Table** **1** displays the lethal concentration (LC) 10, LC25, NOEC (no observed effect concentration), and LOEC (lowest observed effect concentration) values of the SnBr_2_, PbI_2_, and BiAc_3_ precursors. No toxicity at 96 hpf was observed for Cs_2_CO_3_ at any of the conditions tested, and therefore those parameters could not be determined. The concentration‐effect curves for SnBr_2_, PbI_2,_ and BiAc_3_ represent the fraction of surviving embryos (Figure 6b–d). This fraction was determined at each time point by calculating the ratio of the number of live embryos to the total number of embryos treated with the precursors. The results demonstrated a concentration and time‐dependent effect for all the compounds tested. Bi(Ac)_3_ and PbI_2_ were the most toxic compounds and only at the lowest concentration tested (0.01–10 µm) did the fraction of surviving embryos exceed 0.9, while the highest concentrations (20–200 µm) and longer exposure times correlated with a significantly reduced survival fraction (≤0.6). SnBr_2_ showed toxicity only at the two highest doses tested (100 and 200 µm) at 48–96 hpf. Figure 6e compares the embryo viability after 96 h exposure at the different concentrations tested of Bi(Ac)_3_, PbI_2_, and SnBr_2_. The concentration‐response curves further emphasize the dose‐dependent toxicity, with the lowest doses showing no observable harm, but the highest doses (20, 100, and 200 µm) leading to decreased viability. Bi(Ac)_3_ was the most toxic precursor, followed by PbI_2_ and SnBr_2_. Although the counterions alone were not tested, acetate, iodide and bromide are physiological ions, normally found in the organism as trace elements or metabolites. Notably, SnBr_2_ exhibits higher LC10, LC25, NOEC, and LOEC values compared to PbI_2_ and BiAc_3_, suggesting relatively lower acute toxicity (Table 1). The NOEC and LOEC values for SnBr_2_ were 20 times and 10 times higher, respectively, compared to those found with PbI_2_ and Bi(Ac)_3_. However, the safest precursor among the four tested was Cs_2_CO_3_ because no toxic effect in the FET test was detected at concentrations ≤200 µm, in agreement with the cell viability experiments.

**Table 1:** LC10, LC25, NOEC, and LOEC estimate for nanocrystal (NC) precursors: SnBr_2_, PbI_2_ and Bi(Ac)_3_.

Compound	LC_10_ [µm]	LC_25_ [µm]	NOEC [µm]	LOEC [µm]
SnBr_2_	84.521	148.519	20	100
PbI_2_	5.751	44.211	1	10
Bi(Ac)_3_	4.761	33.274	1	10

LC_10_: lethal concentration at which 10% of the embryos are affected; LC_25_: lethal concentration at which 25% of the embryos are affected; NOEC: no observed effect concentration; LOEC: lowest observed effect concentration. All the values were determined with a 95% confidence interval.

This finding suggests that Pb and Bi may pose a greater risk to aquatic organisms at elevated doses and prolonged exposure, highlighting a safer profile of Sn compared to Pb or Bi. Interestingly, previous studies on zebrafish indicated that SnI_2_ was more toxic than PbI_2_, mainly at acidic pH.^[^ ^5a,b^ ^]^ This discrepancy could be attributed to the bioavailability of the metal ion in the biological matrix and the solvent used, as they used dimethylformamide (DMF), whereas our study used water. Additionally, the high doses of PbI_2_ and SnI_2_ precursors tested could produce significant concentrations of toxic hydrogen iodide (HI), likely the primary contributor to the observed toxicity.^[^ ^10^ ^]^ More studies are needed to compare different precursors for Pb‐based and Pb‐free perovskite NCs to determine the influence of precursor solubility in various physiological and environmentally relevant scenarios.

These studies suggest that heavy metals can affect the survival, development, and reproduction of zebrafish and that their toxicity depends on several factors such as exposure time, concentration, chemical composition, and surface functionalization. The results of these studies can contribute to improving the environmental risk assessment and regulation of heavy metals in aquatic ecosystems.

## 3 Conclusion

The results of the study provide compelling evidence that Sn appears to be a safer and more favorable alternative to Pb in perovskites, while Bi exhibits a pulmonary‐ hepato‐ and hemotoxicity profile similar to that of lead (Pb) when tested in human cells and on zebrafish embryos. Both Bi and Pb displayed an oxidative effect in liver cells, indicating the potential for hepatotoxicity at higher concentrations and a cytotoxic profile in pulmonary and red blood cells. This parallel effect in lungs, liver, and red blood cells raises concerns about the overall safety of Bi as a substitute of Pb. On the other hand, Sn compounds, specifically SnBr_2_, demonstrated a notably safer toxicity profile in both, human cells and in zebrafish embryo tests, inducing toxicity only at high doses. Therefore, Sn may be a suitable metal to replace Pb in the synthesis of perovskite NCs for the development of optoelectronic devices with a more environmentally friendly profile. This work provides a basis for understanding the cytotoxicity effects of perovskite NCs in human health and environment, however, more studies are needed regarding the comparison of toxicity of Pb and Pb‐free perovskite materials under different conditions to define a regulatory framework for perovskite NCs development, manipulation, and commercialization.

Ethical Statement

The hemocompatibility study was conducted in accordance with the Declaration of Helsinki and approved by the Xunta de Galicia Ethical Committee (Ref. 2018/369, 19 June 2018). Informed consent was obtained from all subjects involved in the study.

## Conflict of Interest

The authors declare no conflict of interest.

## Supporting information



Supporting Information

## Data Availability

The data that support the findings of this study are available from the corresponding author upon reasonable request.
