# Supplementary material for: The Toxicity of Lead and Lead‐Free Perovskite Precursors and Nanocrystals to Human Cells and Aquatic Organisms
Source: Adv Sci (Weinh). 2025 Feb 10;12(13):2415574. doi: 10.1002/advs.202415574 (PMC11967815; doi:10.1002/advs.202415574)
Supplement: Supplementary file 1 — Supporting Information [file ADVS-12-2415574-s001.docx]

Supporting Information

**The Toxicity of lead and lead-free Perovskite Precursors and Nanocrystals to human cells and aquatic organisms**

Immacolata Maietta, Clara Otero-Martínez, Sabela Fernández, Laura Sánchez, África González-Fernández, Lakshminarayana Polavarapu* and Rosana Simón-Vázquez*

**Experimental Procedures**

**Materials:** Cesium carbonate (Cs_2_CO_3_, 99,9%), cesium acetate salt (CsCH_3_CO_2_, 99.9%), silver acetate salt (Ag· CH_3_CO_2_, 99%), bismuth acetate salt (Bi(CH_3_CO_2_)_3_, 99.9%), lead (II) bromide (PbBr_2_, >98%), lead (II) iodide (PbI_2_, 99%), 1-octadecene (ODE, C_18_H_36_, 90%) oleic acid (OA, C_18_H_34_O_2_, 90%) and oleylamine (OLA, C_18_H_37_N, 70%) were purchased from Merck. Methyl acetate (CH_3_COOCH_3_, 99%) and toluene (C_7_H_8_, <99.8%) was supplied by Alfa Aesar. All chemicals were used without further purification.

**Nanocrystals (NCs) synthesis**

Perovskite NCs were synthesized according to previously reported procedures with slight modifications.^1, 2^

**CsPbBr_3_ and CsPbI_3_ NCs.** In a typical synthesis, a Cs-OL solution is prepared first by loading 1.25 mmol of Cs_2_CO_3_, 1.25 mL of oleic acid, and 20 mL of octadecene in a 40 mL vial. Then, the solution is degassed for 1 hour at 120 ^o^C. On the other hand, a PbX_2_ solution is prepared by loading 0.188 mmol of the corresponding PbX_2_ salt, 5 mL of octadecene, 0.5 mL of oleylamine, and 0.5 mL of oleic acid in a 40 mL vial. The solution is degassed for 1 hour at 120 °C under stirring. Then, the PbX_2_ solution is heated up to 190 °C (for CsPbBr3 NCs) and 175 °C for CsPbI3 under N_2_ and vigorous stirring, and 400 μL of the Cs-OL solution previously preheated at 150 °C is swiftly injected. After 5 s, the reaction is quenched in an ice-water bath. Then, 2.5 ml of MeOAc was added to the NCs crude solution and the NCs were purified by centrifugation (6000 rpm, 5 min). Finally, the supernatant was discarded and the sediment was redispersed in 2 mL of hexane.

**Cs_2_AgBiBr_6_ NCs.** In a typical synthesis, 0.71 mmol of CsOAc, 0.5 mmol of AgOAc, 0.5 mmol of Bi(OAc)_3_, 10 mL of octadecene, 2.5 mL of oleic acid and 0.5 mL of oleylamine are loaded in a 40 mL vial. The solution is degassed for 45 min at 110 °C. For the NCs synthesis, the brown solution is heated up at 180 °C under N_2_ and vigorous stirring and 2.7 mmol of TMSBr is swiftly injected. After 5 s, the reaction is quenched in an ice-water bath. Then, 2.5 ml of MeOAc was added to the NCs crude solution and the NCs were purified by centrifugation (6000 rpm, 5 min). Finally, the supernatant was discarded and the sediment was redispersed in 2 mL of hexane.

For the cell testing, the NCs were then dried with an N_2_ flow to obtain their powder form and dissolved in osmosis water.

**NCs characterization**

UV-Vis extinction spectra were carried out using a Cary-60 UV-Vis spectrophotometer (Agilent). Photoluminescence spectra were obtained with a Cary Eclipse Fluorescence Spectrophotometer (Agilent). Quartz cuvettes with an optical path length of 1 cm were used for both optical analyses. Transmission electron microscopy (TEM) images were obtained with a JEOL JEM 1010 transmission electron microscope operating at an acceleration voltage of 100 kV.

***Cell culture***

Human lung (A549 and NCI-H460) and liver (HEPG2) epithelial cell lines were obtained from American Type Culture Collection (ATCC) and cultured at 37^o^C with 5% CO_2._ The cells were sub-cultured every 2–3 days.

A549 and NCI-H460 cell lines were cultured in Roswell Park Memorial Institute medium (RPMI 1640 medium), while HEPG2 in Dulbecco's Modified Eagle Medium (DMEM; Corning, AZ, USA). All media were supplemented with 10% fetal bovine serum (FBS) (Merck, Darmstadt, Germany), 100 U/mL penicillin and 100 μg/mL streptomycin. In the case of HEPG2, the medium also contained an additional 1 mM sodium pyruvate (Gibco, ThermoFisher Spain).

***MTS cell proliferation colorimetric assay***

Cells were seeded in 96-well plates at densities of 6×10^3^ or 2.5×10^3^ (A549), 5×10^3^ or 2×10^3^ (NCI-H460), 1.5×10^4^ or 1×10^4^ cells/well (HEPG2) for 48 h or 96 h cell viability experiments, respectively. Cells were rested for 24 h (A549 and NCI-H460) or 48 h (HEPG2) to allow adhesion to the well surface and then incubated with different doses of precursors and NCs. Medium alone, medium with precursors and NCs, and untreated cells were used as background and negative controls, respectively. After 48 or 96 h of incubation, a colorimetric cell viability assay was performed using the Cell Titer 96® AQueous One Solution Cell Proliferation Assay kit (MTS, Promega, WI, USA). Plates were further incubated for 2 h with the MTS reagent and the absorbance was measured at 490 nm on a plate multidetector reader (Envision, Perkin-Elmer Inc, CT, USA). Cell viability, expressed as a percentage, was calculated as follows:

*% viability = ([A]perovskite / [A]control) × 100*, where

[A]perovskite represents the absorbance of the cells incubated with the precursors or the NCs minus the absorbance of the medium with precursors or NCs, and [A]control represents the absorbance of the untreated cells minus the absorbance of the culture medium alone.

***Real time cell analysis (RTCA) with the xCELLigence system***

A RTCA was conducted with the xCELLigence system (Agilent, USA), by seeding A549 or HEPG-2 cells in 16-well E-plates (ACEA, Biosciences, Agilent, USA), containing a gold electrode in the base to measure changes in the impedance induce by cell attachment and detachment. Cells were seeded at densities of 2.5×10^3^ (A549) and 1×^104^ cells/well (HEPG-2), respectively, and allow to rest as described in the MTS cell viability assay. Then A549 and HEPG-2 cells were incubated with 100 µM and 200 µM, respectively, of precursors and NCs, due to the different cell sensitivity. The cell index (CI) was recorded for more than 96 h after the addition of the samples. Medium alone, medium with precursors or NCs, and untreated cells were used as background and negative controls. The analysis of the cell viability was performed as mentioned before for the MTS assay at 24, 48, 72 and 96 h.

***Live-dead cell assesment by fluorecent microscopy***

Cells were seeded in 96-well plates at densities of 4x10³ (NCI-H460 and A549) and 7x 10³ (HEPG-2) cells/well. After 24 hours, cells were treated with 500 µM of the precursor (Bi(Ac)_3_, PbI_2_, PbBr_2_, SnBr_2_ and Cs_2_Co_3_) and the NCs (Cs_2_AgBiBr_6_, CsPbI_3_ and CsPbBr_3_) for 96 hours. Cell viability was assessed using a fluorescent-based LIVE/DEAD assay (Invitrogen, USA). Cells were incubated with calcein-acetoxymethyl (calcein-AM, indicator of live cells, green fluorescence) and ethidium homodimer-1 (indicator of dead cells, red fluorescence) for 30 minutes at 37°C. Fluorescence was visualized with a Nikon Eclipse Ti microscope and representative images of each well were taken with the NIS-Elements imaging software (Nikon Instruments Inc., USA).

***Haemolysis***

To analyse the potential haemolytic effect of the NCs and the precursors, we used fresh human whole blood samples from healthy volunteers. The samples and informed consent were obtained following the procedure approved by the Galician Ethical Committee (registry number: 2018/369), in accordance with the guidelines of the Spanish Government.

The blood was diluted 1:2 with phosphate buffered saline (PBS) and centrifuged at 2000 rpm for 10 minutes at 4 ^o^C. Then, the pellet was weighed and resuspended in PBS to a final 3% w/v concentration. Subsequently, 80 μL per well of the blood suspension and the same volume of precursors or NCs in PBS were placed on a 96-well round bottom plate and incubated for 4h at 37 ^o^C. Triton 0.1% and PBS were used as positive and negative controls for haemolysis, respectively. The plate was centrifuged at 2000 rpm for 10 minutes at 4 ^o^C, and aliquots of 80 μL per well of the supernatants were transferred into a 96-well flat-bottom plate. The absorbance of free haemoglobin was measured at 570 nm. Haemolysis, expressed as a percentage, was calculated as follows:

*% Haemolysis = ([A]perovskite - [A]PBS / [A]positive control - [A]PBS) × 100*

where: [A]perovskite represents the absorbance of the cells incubated with any of the compounds (NCs or precursors); [A]PBS represents the absorbance of the cells in PBS (negative control); and [A]positive control represents the absorbance of the cells treated with Triton 0.1%, a surfactant that induces a complete haemolysis of the sample.

***Detection of reactive oxygen species (ROS) release by flow cytometry***

For the measurement of intracellular ROS levels induced by the compounds in A549 or HEPG2 cells, we used the ROS-sensitive probe 2',7'-dichlorodihydrofluorescein diacetate (H_2_DCFDA) (Invitrogen, ThermoFisher Spain). H_2_DCFDA was dissolved in dimethyl sulfoxide (DMSO) to obtain a stock solution with a concentration of 20 mM.

A549 cells were seeded at densities of 4×10^5^, 2.4×10^5^ and 1.2×10^5^ cells per well in 96-well plates for ROS measurement at 24 h, 48 h and 72 h, respectively. Cells were allowed to rest before the addition of 1 μM and 100 μM of the precursors or NCs. After the designated incubation times with the compounds, H_2_DCFDA was added to achieve a final concentration of 2.5 µM, and the plate was incubated for 30 min at 37 °C. Propidium iodide (PI) was also added at a concentration of 250 µg/mL to distinguish between live (IP^-^) and dead (IP^+^) cell populations. Phorbol 12-myristate 13-acetate (PMA) was used as positive control at a concentration of 10 ng/mL (Alfa Aesar, ThermoFisher, Spain). The samples were measured on a BD Accuri™ C6 Flow Cytometer (1×10^4^ events/sample) and the data analysed using the BD Accuri™ C6 Software (BD Biosciences, NJ, USA).

***TEM microscopy***

A549 cells were incutabet with 100 μM of the NCs for 96 h. The cells were fixed with 2.5% glutaraldehyde in 0.05M cacodylate buffer for 3 h at 4ºC. After three washes with 0.1 M cacodylate buffer, the cells were incubated with 1% tannic acid in 0.1 M cacodylate buffer for 1 h at 4ºC. The samples were dehydrated using a graded ethanol concentration and then embedded in Epon resin. Then, they were cut into 70 nm sections using an ultra-microtome in sections and examined with a transmission electron microscope (JEM-1010) (Peabody, MA, USA).

***Statistical analysis***

Graphpad Prism version 8 for Windows (GraphPad Software, CA, USA) was used for statistical analysis and graph representation. All data are expressed as the mean ± standard deviation (SD) from three independent experiments. A two-way analysis of variance (ANOVA) test was conducted to determine the statistical significance of the differences between cells incubated with NCs or the precursors and untreated cells for *in vitro* data. A p-value of < 0.05 was considered as significant. P-values of ≤0.03, ≤0.002, ≤0.0002, ≤0.0001 are indicated as single (*), double (**), triple (***) and quadruple (****) asterisks, respectively.

***Zebrafish embryos: handling and care***

For *in vivo* studies of the toxicity of NC precursors, the zebrafish model was utilized, an emerging model with several advantages over other *in vivo* models. Zebrafish embryos are commonly employed in early life-stage tests, particularly for investigating the toxicity and teratogenicity of chemicals. ^3^  Wild-type Zebrafish (Danio rerio) were housed in recirculating reverse osmosis water systems with a pH of 7± 0,5, a conductivity of 650 µS/cm, and a temperature of 26 ± 2 ºC in a room with a 14:10-hour light:dark photoperiod.^4^

Adult fish were fed twice daily with artemia (saline artemia) and a high-protein compound (Gemma Micro 300). Adult male fish (odd number) were crossed with females to obtain embryos through natural spawning. The collected embryos were placed in petri dishes with osmosis water and examined under a microscope (Nikon TMS) to select the viable ones.

The maintenance of wild zebrafish was carried out in the approved animal facility of the University of Santiago de Compostela (authorized with the REGA code ES270280346401) in accordance with Spanish standard protocols regarding animal care (Directive 2012-63-UE). At the end of the experiment, the embryos were euthanized with an overdose of tricaine (Sigma).

***In vivo toxicity assessment using zebrafish embryos***

For the toxicity studies, we conducted the official Fish Embryo Acute Toxicity (FET) test (OECD 2013). Viable embryos (0-4 hpf) were carefully selected and incubated with different concentrations of NC precursors dissolved in osmosis water, alongside corresponding controls, in 96-well plates for a duration of 96 h.

The plates were then placed in an incubator at a temperature of 26 ºC with a photoperiod of 14 hours of light and 10 hours of darkness. Each concentration was tested with 20 embryos, and 32 embryos were used as negative control (left untreated). As a positive control we employed 3,4-dichloroaniline at 100%.

Embryos were observed under an inverted optical microscope (Nikon TMS) at 24, 48, 72 and 96 hours of treatment. To assess embryo toxicity, microscopic observations focused on the indicators specified in the FET test: embryo coagulation, absence of somite formation, absence of separation of the yolk tail and absence of a heartbeat.

The toxicity results of the FET assays were obtained using a statistical analysis program designed for biological tests known as ToxRat (ToxRat Solutions. 2003. ToxRat® Alsdorf, Germany), following OECD guidelines. The program analysed the effects of various concentrations of NC precursors on embryo survival. The values of the lethal concentration for 10% (LC10) or 25% (LC25), the No Observed Effect Concentration (NOEC) and the Lowest Observed Effect Concentration (LOEC) were calculated based on accumulated mortality data for each exposure period (24,48,72 and 96 h) using probit analysis.

**Table S1.** Half-inhibitory concentration (IC50) of the NCs and precursors in HEPG-2, NCI-H460 and A549 cells after 96 h of incubation determined by the dose-curve response of the MTS assay in Figure 3a-c.

| **IC_50_ (µM)** | **HEPG-2** | **NCI-H460** | **A549** |
| --- | --- | --- | --- |
| Bi(Ac)_3_ | 500-1000 | 217 | 656 |
| PbI_2_ | 500-1000 | +1000 | 725 |
| PbBr_2_ | 500-1000 | 596 | 680 |
| SnBr_2_ | +1000 | 843 | +1000 |
| Cs_2_Co_2_ | +1000 | +1000 | +1000 |
| CsPbI_3_ | 500-1000 | +1000 | 883 |
| Cs_2_AgBiBr_6_ | +1000 | 182 | 161 |
| CsPbBr_3_ | +1000 | +1000 | +1000 |

**
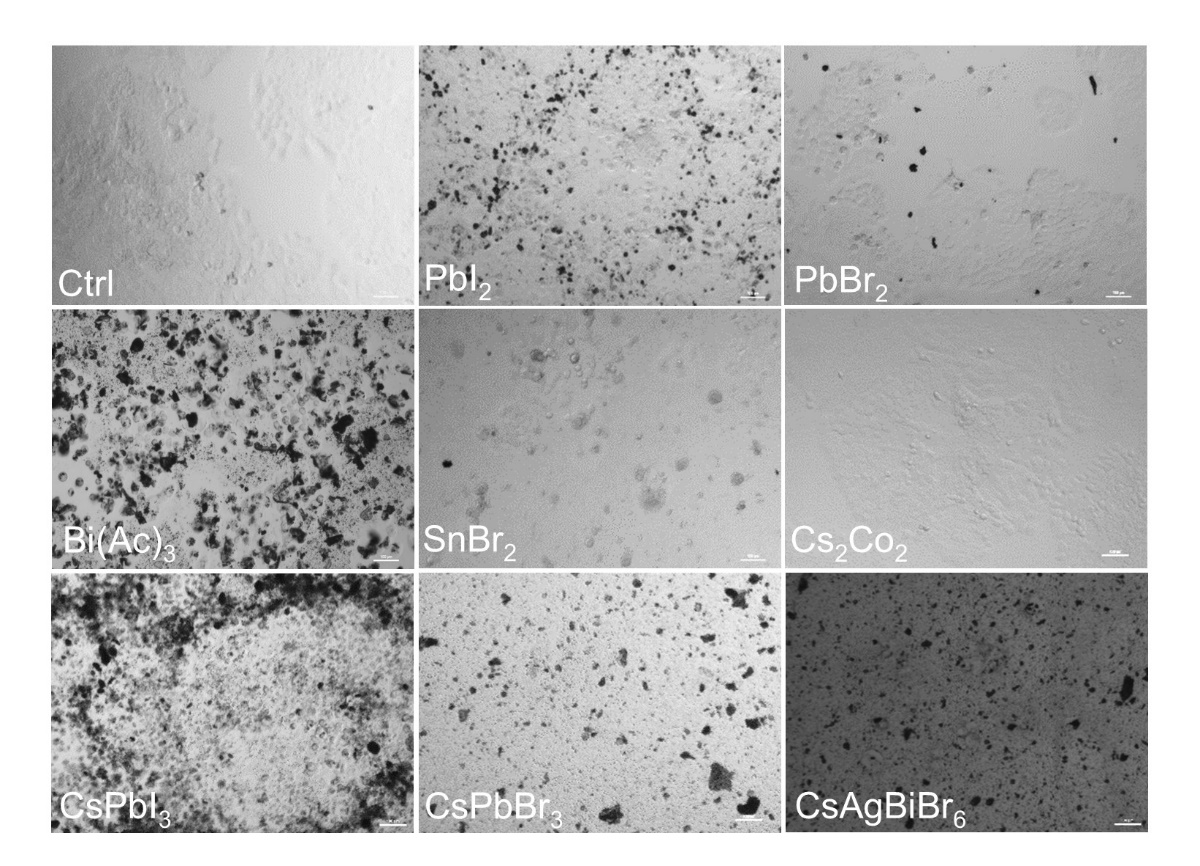
**

**Figure S1**. Aggregates of perovskite precursors and NCs at 1 mM in NCI-H460 cells. The images were taken at a magnification of 10×. Scale: 100 µm.


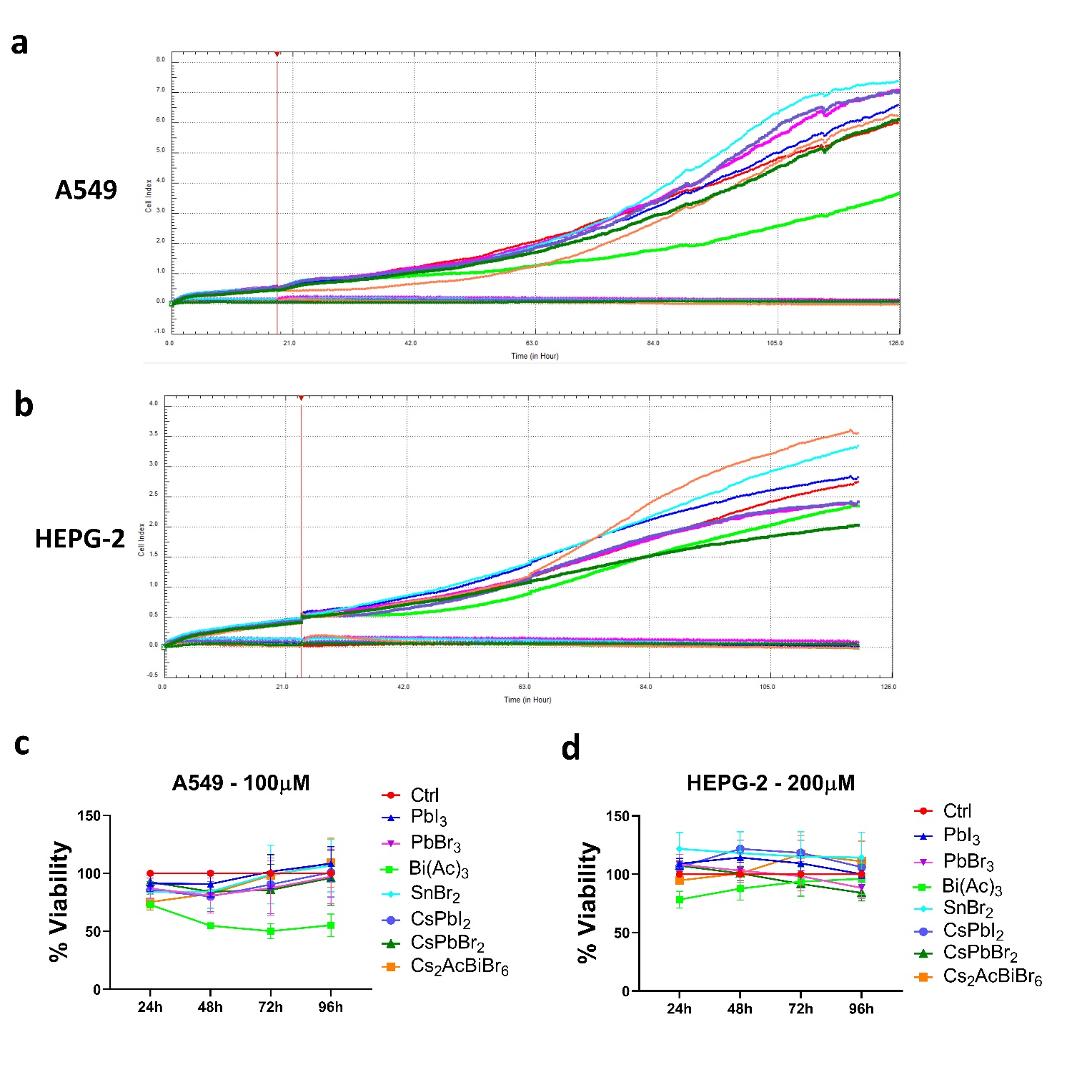
**Figure S2**. Real-time changes in the cell index determined by the xCELLigence system in A549 (a) and HEPG-2 cells (b) induced by perovskite precursors and NCs at 100 µM and 200 µM, respectively. Kinetics of cell viability at 24, 48, 72, and 96 h for A549 (c) and HEPG-2 cells (d).


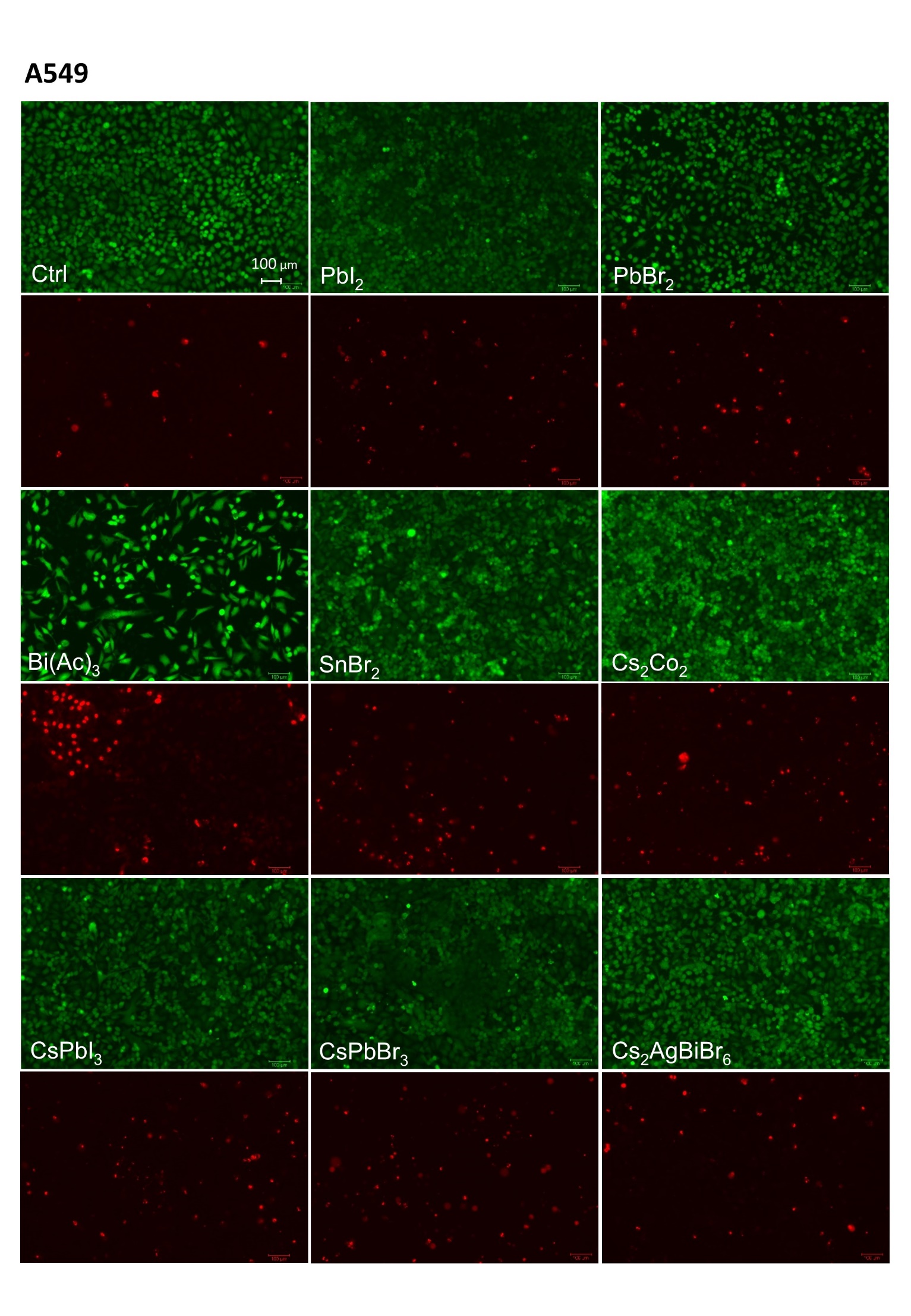

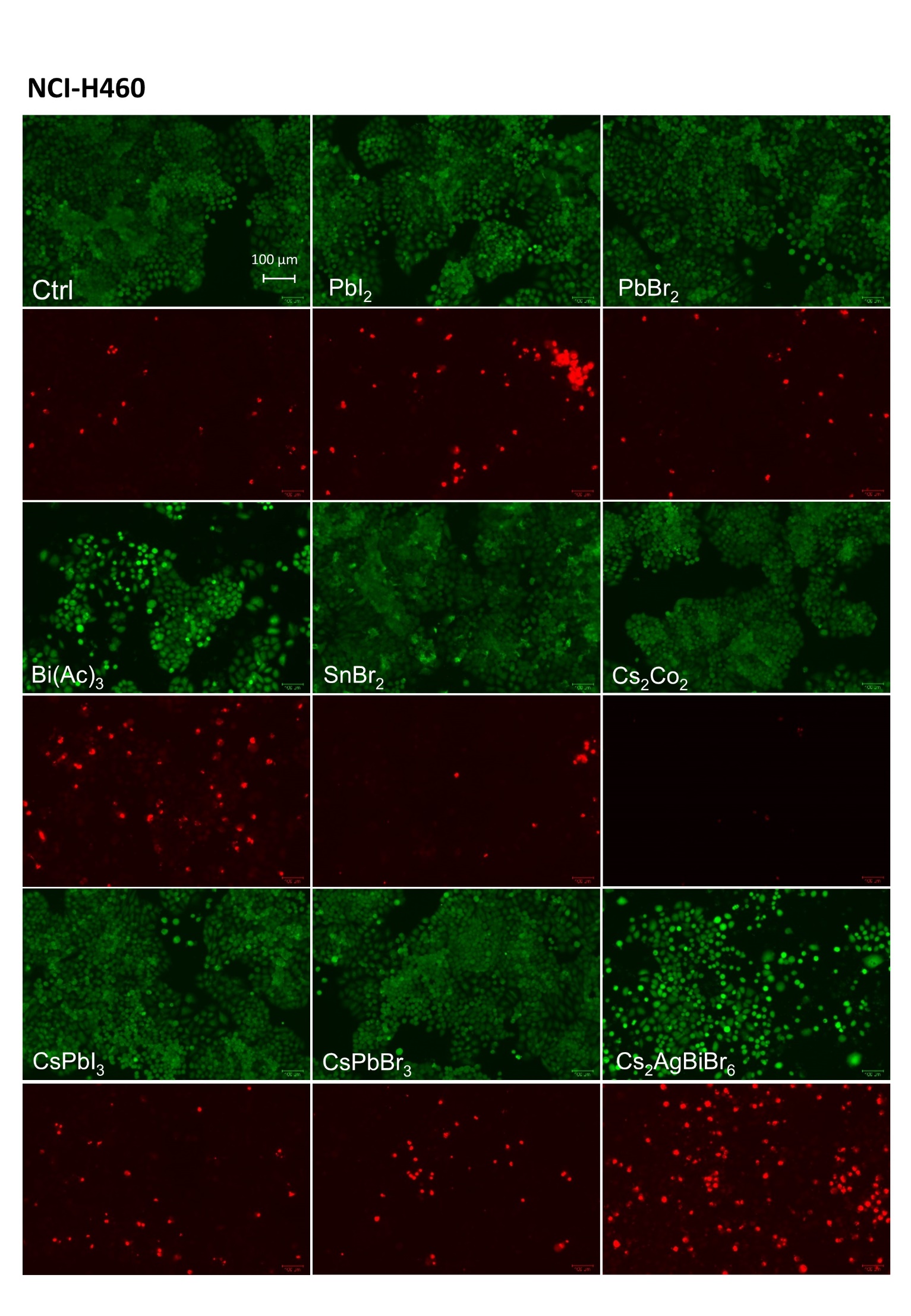

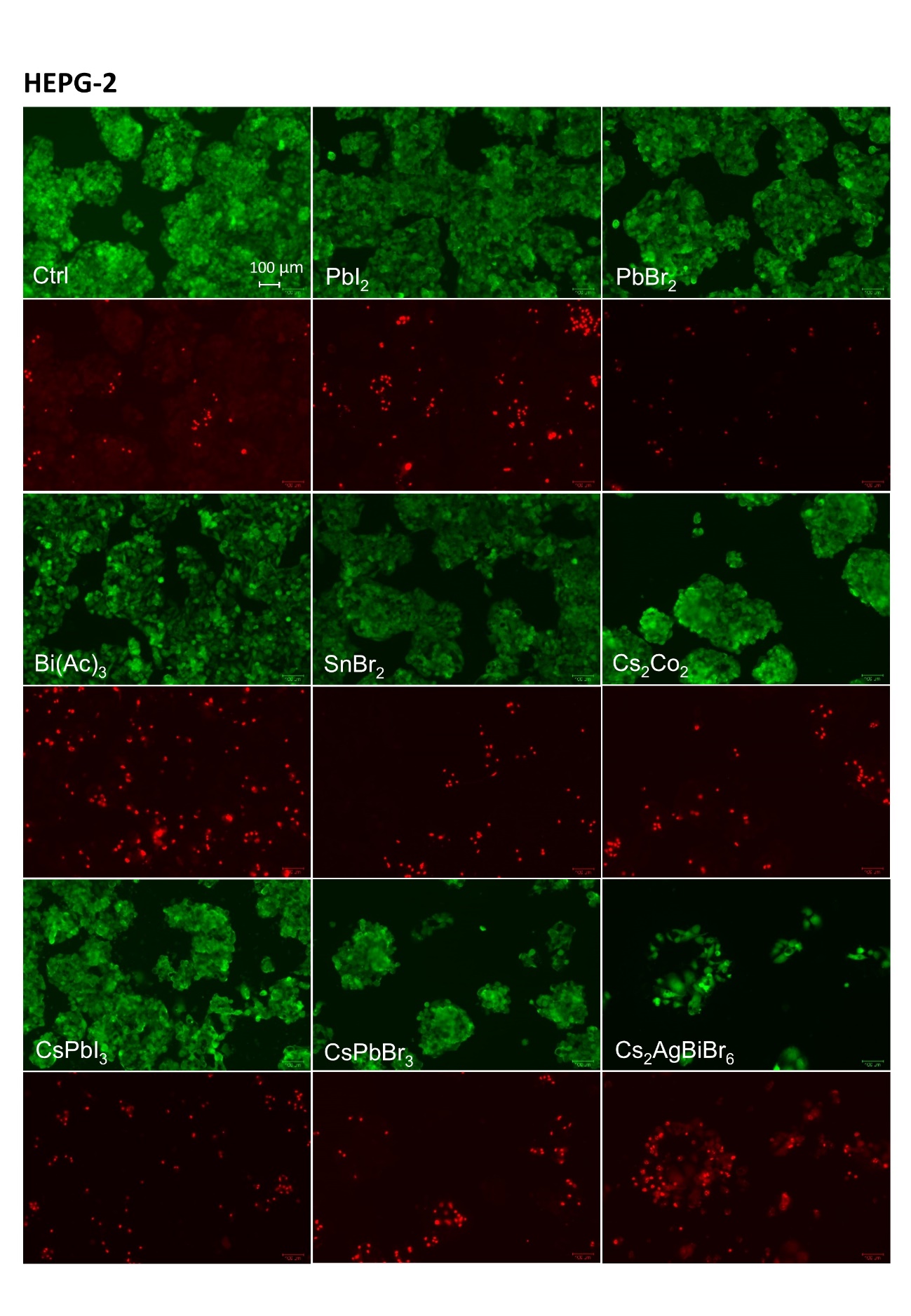


**Figure S3**. Fluorescence images of the A549, NCI-H460, and HEPG-2 cells incubated with the perovskite precursors and NCs at 500 µM for 96 h and labeled with calcein-AM (green) and ethidium homodimer-1 (red). The white bar represents the scale:100 µM.


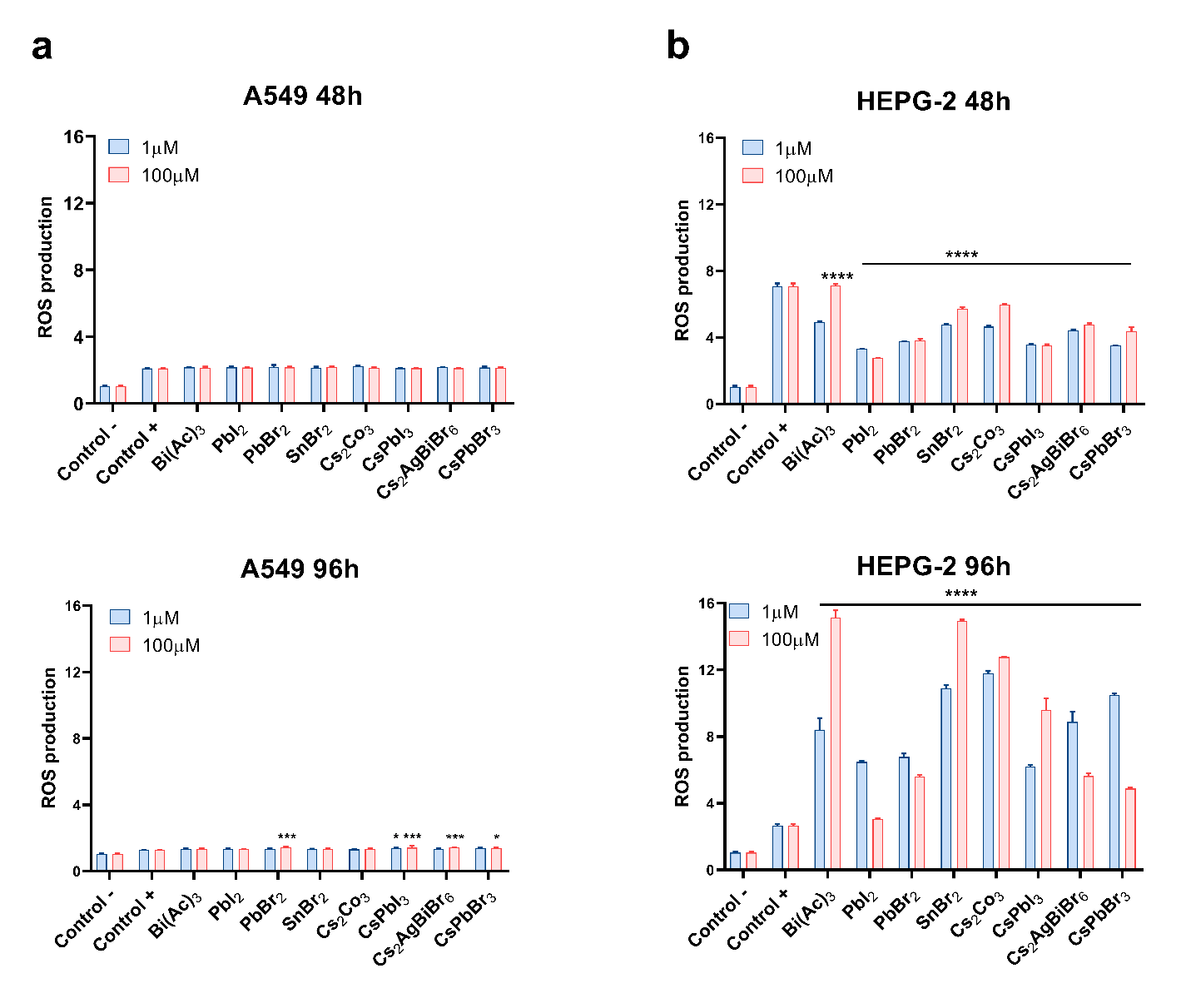


**Figure S4**. Quantification of Reactive oxygen species (ROS) production by flow cytometry. Intracellular ROS production by (a) A549 and (b) HEPG-2 cell lines after incubation with the perovskite precursors Bi(Ac)_3_, PbI_2_, PbBr_2_, SnBr_2,_ and Cs_2_CO_3_, as well as the perovskite NCs Cs_2_AgBiBr_6_, CsPbI_3_ and CsPbBr_3_ at different concentrations (1 and 100 μM) and time points (48 and 96 hours). The SnBr_2_ precursor was also tested for comparison. The mean fluorescence intensity was normalized to the basal ROS production in untreated cells and calculated using Graphad Prism 8. Phorbol 12-myristate 13-acetate (PMA) was used as positive control. The presented data, representing mean ± SD from three experiments, indicate significant differences compared to the positive control (* p= 0.0332; ** p= 0.0021; *** p=0.0002; **** p <0,0001).

**References**

1. Protesescu, L.; Yakunin, S.; Bodnarchuk, M. I.; Krieg, F.; Caputo, R.; Hendon, C. H.; Yang, R. X.; Walsh, A.; Kovalenko, M. V., Nanocrystals of Cesium Lead Halide Perovskites (CsPbX3, X = Cl, Br, and I): Novel Optoelectronic Materials Showing Bright Emission with Wide Color Gamut. *Nano Letters* **2015,** *15* (6), 3692-3696.

2. Creutz, S. E.; Crites, E. N.; De Siena, M. C.; Gamelin, D. R., Colloidal Nanocrystals of Lead-Free Double-Perovskite (Elpasolite) Semiconductors: Synthesis and Anion Exchange To Access New Materials. *Nano Letters* **2018,** *18* (2), 1118-1123.

3. Zhang, W.; Lin, K.; Miao, Y.; Dong, Q.; Huang, C.; Wang, H.; Guo, M.; Cui, X., Toxicity assessment of zebrafish following exposure to CdTe QDs. *Journal of Hazardous Materials* **2012,** *213-214*, 413-420.

4. Westerfield, M. J., The Zebrafish Book; A guide for the laboratory use of zebrafish (Danio rerio). **2007**.
